# Metabolomic study of soft corals from the Colombian Caribbean: PSYCHE and ^1^H-NMR comparative analysis

**DOI:** 10.1038/s41598-020-62413-0

**Published:** 2020-03-25

**Authors:** Liliana Santacruz, Diana X. Hurtado, Roisin Doohan, Olivier P. Thomas, Mónica Puyana, Edisson Tello

**Affiliations:** 1 Bioprospecting Research Group and Biosciences Doctoral Program, Faculty of Engineering, Campus Puente del Común, Universidad de la Sabana, 250001 Chía, Colombia; 2 Marine Biodiscovery, School of Chemistry and Ryan Institute, National University of Ireland Galway (NUI Galway), University Road, H91 TK33, Galway, Ireland; 3 Departamento de Ciencias Biológicas y Ambientales, Universidad Jorge Tadeo Lozano, Carrera 4 # 22-61, 110311 Bogotá, Colombia

**Keywords:** Computational biology and bioinformatics, Cheminformatics

## Abstract

Marine organisms have evolved to survive against predators in complex marine ecosystems via the production of chemical compounds. Soft corals (Cnidaria, Anthozoa, Octocorallia) are an important source of chemically diverse metabolites with a broad spectrum of biological activities. Herein, we perform a comparative study between high-resolution proton nuclear magnetic resonance (^1^H-NMR) and pure shift yielded by chirp excitation (PSYCHE) experiments to analyze the metabolic profile of 24 soft corals from the Colombian Caribbean to correlate chemical fingerprints with their cytotoxic activity against three cancer cell lines (human cervical carcinoma (SiHa), human prostatic carcinoma (PC3) and human lung adenocarcinoma (A549)). All data obtained were explored using multivariate analysis using principal components analysis (PCA) and orthogonal partial least squares (OPLS) analysis. The results did not show a significant correlation between clusters using ^1^H-NMR data in the PCA and OPLS-DA models and therefore did not provide conclusive evidence; on the other hand, a metabolomic analysis of PSYCHE data obtained under the same parameters revealed that when a decoupled experiment is performed, it was possible to establish a statistically valid correlation between the chemical composition of soft corals and their cytotoxic activity against the PC3 cancer cell line, where the asperdiol and plexaurolone markers were putatively identified and related to the cytotoxic activity presented by extracts of *Plexaurella* sp. and *Plexaura kukenthali*, respectively. These results increase the speed, effectiveness and reliability of analyses for the study of this type of complex matrices.

## Introduction

Metabolomics studies allow a complete analysis of a set of metabolites that are the substrates and products of metabolism driving essential cellular functions in a given biological system^1^. This research has applications in different fields, such as pharmacology, environmental sciences, chemotaxonomy, nutrition and medicine^2^. Recently, metabolomic approaches have allowed the understanding of complex biological systems and the biochemical composition of organisms that live in diverse environments, such as marine areas^3^. Goulitquer *et al*. demonstrated that metabolites are important links between genotype and phenotype and are important for studying several biological processes and for analyzing interactions between organisms within communities via mass spectrometry (MS)-based metabolomics^4^. In addition, Mohamed A. Farag *et al*. compared metabolomics results obtained with liquid chromatography coupled to mass spectrometry (LC-MS) with those obtained by nuclear magnetic resonance (NMR) to investigate the metabolism of 16 Sarcophyton species in the context of their genetic diversity and growth habitats^3^.

The importance of studying marine invertebrates lies in their extraordinary ability to produce a broad variety of chemical compounds with unique chemical structures that in most cases have been correlated with significant biological activities, which has led to the successful development of commercial drugs. One of the best-known compounds is ziconotide (Prialt®), a synthetic derivative of a peptide originally isolated from the venom of the marine snail *Conus magus;* this compound is a neuronal calcium antagonist useful for treating severe chronic pain^5^. Trabectedin ET-743 (Yondelis^TM^), a complex peptide originally isolated from *Ecteinascidia turbinata*, is effective in the treatment of many types of cancer, including melanoma, sarcoma, and lung, breast, ovarian, endometrial and prostate cancer^6^. Brentuximab vedotin®, originally isolated from the marine opisthobranch *Dolabella auricularia*, is used against breast and Hodgkin’s lymphomas^7^. Eribulin mesylate (Halaven®), originally isolated from the marine sponge *Halichondria okadai*, is a potent microtubule inhibitor used in the treatment of breast cancer^8^, and vidarabine (Vira-A®), a modified nucleoside originally isolated from the Caribbean sponge *Cryptotethya crypta*, is an inhibitor of viral DNA polymerases and other enzymes and is used against varicella zoster and herpes viruses^9^. This information allows us to infer that marine organisms are a prolific source of bioactive and novel molecules that can be used as potential agents against different human illnesses.

Bioprospecting marine research in the Colombian Caribbean has established organisms with various biological activities, mainly anti-inflammatory, cytotoxic and antiviral activities, e.g., gorgonian *Antillogorgia (Pseudopterogorgia) elisabethae* collected at the Islands of Providencia and San Andrés showed anti-inflammatory properties^10^; additionally, the octocorals *Eunicea laciniata* and *Eunicea asperula* have been evaluated for their cytotoxic and antiviral activities, where dolabellane diterpenes isolated from the soft corals *E. laciniata* and *E. asperula* showed anti HSV-1 activity^11^. In addition, the prostaglandins isolated from the soft coral *Plexaura homomalla* presented anti-inflammatory activity^12^.

There is no single analytical technology or protocol to analyze the overall metabolome of an organism and obtain a complete metabolic profile^13^. However, metabolomics approaches use hyphenated and high-throughput techniques to perform chromatographic separation of metabolites using either liquid chromatography (LC) or gas chromatography (GC) coupled with mass spectrometry or nuclear magnetic resonance (NMR) to analyze complex mixtures of metabolites from different organisms^14^. Furthermore, each technique has advantages and drawbacks, and although the analytical technique most often used for metabolite profiling is liquid chromatography-mass spectrometry, due to its high sensitivity and the wide range of molecules that can be analyzed, the use of NMR experiments has expanded rapidly over the past ten years^15^. Regardless of the low sensitivity of NMR, this technique presents advantages, such as being nondestructive and requiring no sample preparation and relatively short acquisition times. NMR is a highly reproducible method for metabolomics studies. At present, it is possible to record NMR spectra from crude extracts^16^ to perform preliminary studies of the metabolic composition of marine organisms. NMR analyses give a global overview of all metabolites present in complex biological samples such as soft coral extracts that produce compounds such as prostaglandins, sterols and a wide range of terpenes, and these compounds represent the main chemical defense of these organisms against predators^17,18^. On the other hand, metabolomic studies using ^1^H-NMR on mixtures may experience signal overlap, especially in samples that contain significant amounts of fatty acids and terpenoids, hampering comparative metabolomic studies of extracts from soft corals^19,20^. The Table S1 in Supplementary Information shows the advantages and disadvantages of the most commonly used methods for metabolomics analysis.

The overlapping signals in ^1^H-NMR experiments are a complex issue, and to greatly improve spectral resolution and the gathering of metabolomics data, new NMR techniques such as Pure Shift Yielded by Chirp Excitation (PSYCHE) are proposed. This method proposed by Morris and coworkers^21^ contains two low flip angle (*β*) swept-frequency pulses in the presence of a weak pulsed field gradient. The advantage of this technique over ^1^HNMR is that it resolves overlapping ^1^H-^1^H scalar coupling multiplets, which improves chemical shift analysis of complex natural products^22^. PSYCHE suppresses the effects of homonuclear coupling and allows observation of decoupled ^1^H-NMR spectra with chemical shifts only, helping in the identification of potential biomarkers in metabolomic studies^16^.

Here, we used ^1^H and PSYCHE NMR experiments to perform a metabolomic comparison of 24 soft coral extracts (complex biological samples) to examine whether there is a correlation between the chemical composition of the extracts and their cytotoxicity against SiHa, PC3 and A549 tumor cancer cell lines^23^. When large data sets are analyzed, a multivariate analysis (MVA) is a valuable approach for the identification of potential key markers in complex mixtures.

Due to this, MVA was developed using the principal component analysis (PCA) algorithm to compress a dataset onto a lower-dimensional feature subspace maintaining most of the relevant information allowing to stablish chemical differences in the NMR experiments^24^. This approach was followed by the orthogonal projection to latent structures discriminant analysis (OPLS-DA), which is a supervised model that filters out orthogonal metabolite variables that are not related to categorical variables to discriminate and separate predictive from nonpredictive (orthogonal) variation. A response matrix Y (containing toxicity data) was correlated to a descriptor matrix X (containing spectral data) that is orthogonal (noncorrelated) to Y to identify the markers that contributed to the discrimination of cytotoxic activity and to determine outliers^25^. The reliability of the model was verified by the cross validation method, and the parameters for the OPLS model, R2 and Q2, were calculated (varying from 0 to 1), where R2 corresponded to the fraction of the variance explained by the model, Q2 suggests the predictive capability of the model^26^, and it cross validation reproducibility Q2/R2, was also considered indicative of relevant associations^27^.

Furthermore, the variable importance in projection (VIP) was calculated by using partial least squares-discriminant analysis (PLS-DA) model^28^, which describes a quantitative estimation of the discriminatory power of each individual feature and summarizes the contribution a variable makes to the model^29^. A combination of univariate and multivariate statistical approaches (VIP > 3, p < 0.05) was used as criteria to discriminate the key markers^30^.

OPLS-DA allowed to discriminate the soft coral extracts by separating predictive and nonpredictive data^31^. Finally, MVA and its loading and score plots, which are closely linked such that features (chemical shifts) that are highly loaded in a specific direction in the loading plot contribute to an increased degree to the observations (soft corals) that are located in that direction in the score plot. This allowed us to establish that the key markers B2_5118, B4_4965 and B4_7686 were mainly responsible of the cytotoxic activity exhibit by the extracts of the soft corals *Plexaurella* sp. and *P. kukenthali* against the tumor cancer cell line PC3^23^. This article describes a workflow that helped to estimate the confidence levels of compound annotations in reported metabolites that were used as established by the Metabolomics Society at 2017^32^. In summary, this work achieved a confidence level of 2 for features (VIPs) most responsible for cytotoxic activity considering that the feasible structure was compared with data or databases of literature by diagnostic evidence^33^.

## Results

### ^1^H-NMR metabolomic fingerprints

**Figure 1:**
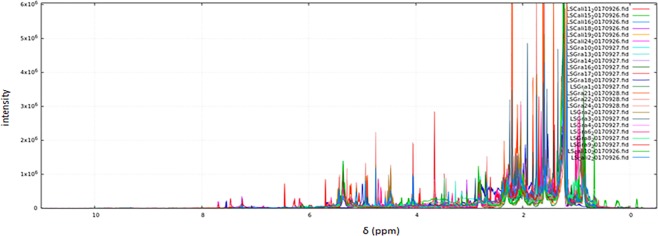
^1^H-NMR data of 24 soft coral extract samples using the open access software NMRProcFlow v1.2 25. Spectra of soft coral extracts are presented in different colors.

Cytotoxic effect correlation between PC3, A549 and SiHa cancer cell lines and chemical composition using ^1^H-NMR spectra of the 24 crude extracts of soft corals (in the genera *Plexaura, Pseudopterogorgia*, *Eunicea*, *Plexaurella* and *Pseudoplexaura* Table S2 in Supplementary Information) were explored over 168 spectral bins (variables) describing metabolic profiles using the open access NMRProcFlow v1.2 software^34^ developed by INRA Science & Impact in France. The complexity of the spectra can be visualized in some regions due to overlapping of signals (Fig. 1). The data processing script is described in the Supplementary Information (Table S4).

**Figure 2:**
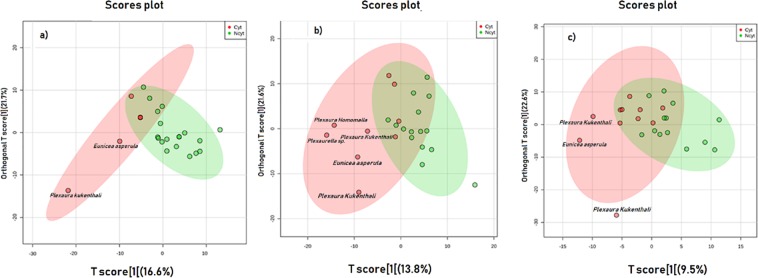
OPLS-DA Score plots of ^1^H-NMR metabolomic data of 24 soft coral extracts based on their cytotoxic activity against three tumor cell lines. (**a**) human lung adenocarcinoma, A549 (**b**) human prostatic carcinoma, PC3 and (**c**) human cervical carcinoma, SiHa. Red dots represent active extracts, and green dots represent extracts that were not active. The ellipse represents the 95% confidence interval of the PLS-DA model^71^.

Exploratory data analysis was performed by PCA using the MetaboAnalyst version 3.0 web application^35^ (Supplementary Fig. S1). For the tumor cell lines A549, PC3 and SiHa, extracts of *P. kukenthali, E. asperula* and *Plexaurella* sp. were most distant from those groups that exhibited the highest cytotoxic activity against the three tumor cell lines. Additionally, we used partial least squares-discriminate analyses (OPLS-DA) (Fig. 2) to visualize increased separation of the groups that presented cytotoxic activity from those that did not. Finally, to demonstrate that the observed PCA was valid, the results from the plots (loading and observations) were contrasted, with the results obtained in the PLS-DA model. Additionally, the statistical parameters for the PLS-DA models for the classification of the ^1^H-NMR data were determined, as shown in Table 1.

**Table 1:** Statistical parameters of PLS-DA models for classification of ^1^H-NMR experiments according to cytotoxic and noncytotoxic groups from 24 extracts of soft corals tested against three tumor cell lines; A549: human lung adenocarcinoma, PC3: human prostatic carcinoma and SiHa: human cervical carcinoma.

PLS-DA parameters			
Cell lines	Q2	R2	Q2/R2
A549	0.14	0.49	0.28
PC3	−0.02	0.49	−0.04
SiHa	0.03	0.59	0.05

The analysis was done using a 10-fold cross-validation method.

Q2: predictive capability, R2: correlation coefficient.

In this study, an extract was considered as active, if it showed inhibition of tumor cell lines ≥40% at 20 μg/mL. This designation included extracts that showed moderate to strong cytotoxic activity^36^ (Table S3).

With the purpose of discriminating between metabolite profiles (analysis of a large group of metabolites which are related to a class of compounds^37^) and cytotoxicity of all the extracts analyzed, a validation of the model was carried out using a PLS-DA based on the PLS algorithm. The discriminating variable was cytotoxic activity.

A cross validation test was developed for the classification model using the three tumor cell lines, the models were analyzed using R2 and Q2 metrics and the Q2/R2 ratio. Predictive relevance is considered when values are greater than 0.5^38^. The resulting models for each tumor cell line revealed an overfitting in the separation of the metabolic profiles. Discrimination of the samples according to their cytotoxic activity is shown in Table 1.

### Pure shift experiments (PSYCHE) in metabolomic fingerprints

**Figure 3:**
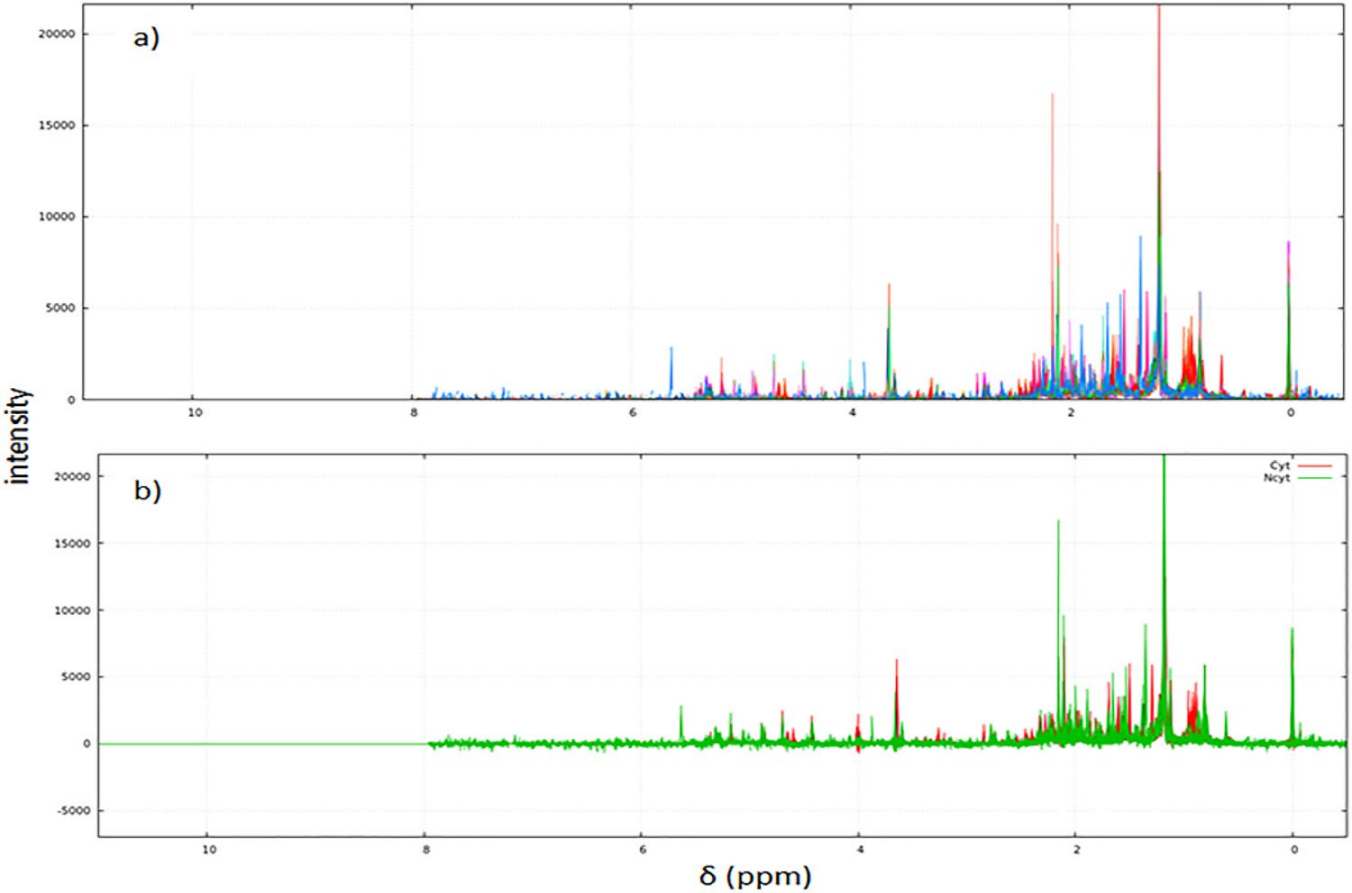
(**a**) PSYCHE spectra of the 24 soft coral extracts analyzed. The spectrum of each soft coral extract is represented in a different color. (**b**) Spectra separated into two groups, by color. Red (cytotoxic extracts), green (not cytotoxic extracts).

To compare the results (cytotoxic activity vs. metabolic profile) between the ^1^H and PSYCHE NMR experiments, the same protocol and the same number of samples were used for both experiments. The PSYCHE data was analyzed over 113 spectral bins (variables) describing metabolic profiles using the NMRProcFlow v1.2 software^34^. The PSYCHE spectra of the 24 soft coral extracts analyzed are shown in Fig. 3a. Figure 3b shows the same 24 spectra but separated into two groups by color: cytotoxic extracts appear in red, and noncytotoxic extracts appear in green. To establish bins responsible for group separation (VIP), it was necessary to perform a statistical analysis to show the relevant chemical shift that allowed the discrimination.

PCA^35^ showed that the cytotoxic extracts of *P. kukenthali* and *E. asperula* are separated in PC2 for the tumor line A549, as shown in Supplementary Fig. S2a. On the other hand, the PC2 shown in the model for the PC3 tumor line showed that cytotoxic extracts of *P. kukenthali*, *E. asperula* and *Plexaurella* sp., were the most separated, which agrees with the results obtained for this same tumor cell line using the ^1^H-NMR experiment (See Supplementary Fig. S2b). Finally, PC1 and PC2 from PCA generated for the SiHa tumor cell line did not show a clear separation between the extracts and their cytotoxic activity, as shown in Supplementary Fig. S2c.

**Figure 4:**
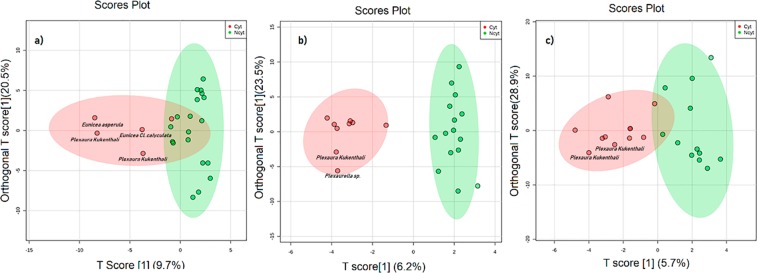
OPLS-DA Scores Plot^35^ of PSYCHE experiment of metabolomics data from 24 soft coral extracts based on their cytotoxicity against three tumor cell lines. (**a**) human lung adenocarcinoma A549, (**b**) human prostatic carcinoma PC3 and (**c**) human cervical carcinoma SiHa. Red dots represent active extracts, and green dots represent extracts that were not active. Red dots represent cytotoxic extracts, green dots represent extracts that were not cytotoxic. An extract was considered active if exhibited inhibition of the tumor cell line ≥ 40% at 20 μg/mL)^72^. The ellipse represents the 95% confidence interval of the model^71^.

It is appropriate to apply PCA as a first step for exploratory studies where differences between experimental groups may be unknown or unpredictable; however, the spectral noise and high within-group variation do not show a separation between groups in many cases^39^; therefore, to overcome this problem, a supervised model OPLS-DA was constructed for classification of the samples and to improve discrimination between metabolic profiles and their cytotoxic activity (Fig. 4) because the OPLS-DA algorithm is normally applied when there are only two classes, improving the class discrimination and robustness of important feature identification^40^.

As performed for the ^1^H-NMR experiments, the validation of the PSYCHE models (Table 2) was carried out using PLS-DA, where the models were evaluated using R2, Q2 and Q2/R2 metrics. A model is considered predictive if the Q2/R2 ratio is greater than 0.5^41^. When the results of the models obtained for the ^1^H-NMR experiments were compared with those from the PSYCHE NMR experiment (Tables 1 and 2), an improvement in the total amount of variance (R2) and in the accuracy (Q2) was observed for the latter experiment. This may be because the soft coral extracts contain many overlapping peaks from multiple compounds and that such overlaps obscure peak assignments and compromise the quantitative analysis, which explains the overfitting observed in the OPLS-DA model using ^1^H-NMR data compared to that in the decoupled PSYCHE model; all this is reflected in the total reproducibility explained by the ratio Q2/R2^42^. However, a reasonable separation between the groups (cytotoxic and noncytotoxic) was only evident in the model generated for the PC3 tumor cell line, with a Q2/R2 value of 0.59 (Table 2). These results confirm that cluster separations in the OPLS-DA score plot shown in Fig. 4b for the PC3 tumor cell line were statistically significant.

**Table 2:** Statistical parameters of PLS-DA models for classification of PSYCHE experiment according to cytotoxic and non-cytotoxic groups from 24 extracts of soft corals tested against three tumor cell lines; A549: human lung adenocarcinoma, PC3: human prostatic carcinoma and SiHa: human cervical carcinoma.

PLS-DA parameters			
Cell lines	Q2	R2	Q2/R2
A549	0.14	0.76	0.19
PC3	0.39	0.66	0.59
SiHa	0.07	0.72	0.09

The analysis was done using a10-fold cross-validation method

Q2: predictive capability, R2: correlation coefficient.

The cytotoxic activity of each extract against the tested tumor cell lines showed that the extracts of *P. kukenthali* (code G18P) and *Plexaurella* sp. (code G22P) exhibited inhibition percentages of 64.0% and 63.5% against the PC3 tumor cell line, respectively. In Fig. 4b, the OPLS-DA of PSYCHE experiments show that those extracts were part of the group that presented cytotoxic activity against the tumor line PC3.

Information on the metabolites responsible for the separation between cytotoxic and noncytotoxic groups (VIP) for the tumor cell line PC3 (statistically significant model) was extracted from the PLS-DA. This process allowed us to identify key discriminatory metabolites through a VIP analysis, which revealed several distinguishing patterns. High values indicate the increased discriminatory power of particular metabolites. Variables with VIP > 1.0 are considered potential biomarker candidates for group discrimination^43^. However, for this study, only variables with VIP > 3.0 were considered because these variables play important roles in the discrimination of cytotoxic activity, as three markers were the major variables responsible for group separation and the chemical shifts corresponding to these VIP markers in the NMR experiments were well resolved, which allowed an improved interpretation of the results.

**Figure 5:**
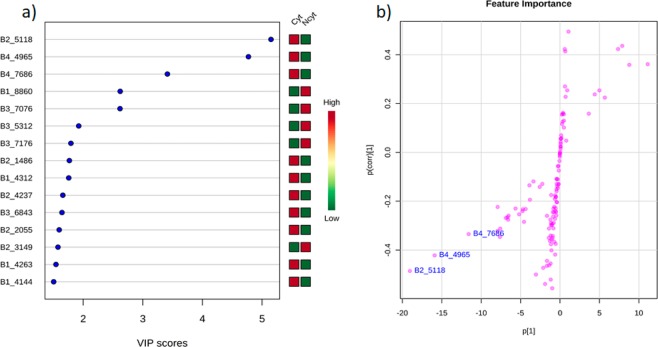
(**a**) VIP (variable importance in the projection) scores/p < 0.05^35^ obtained from the PSYCHE PLS-DA model (**b**). Feature importance from the OPLS S-plot^71^.

The three markers that showed the highest scores in the VIP analysis (Fig. 5a) were B2_5118 (score = 5.1), marker B4_4965 (score = 4.8) and marker B4_7686 (score = 3.5). Additionally, in the OPLS S-plot (Fig. 5b), each of the three markers selected as VIPs can be clearly distinguished, with each coordinate representing a single NMR signal (contributing variables to the classification). This result shows that features (VIP) correlated with extracts of *P. kukenthali* (Gra 18) and *Plexaurella* sp. (Gra 22) have cytotoxic activity against the PC3 tumor cell line, in accordance with the analysis obtained from the OPLS-DA (Fig. 4b).

The previously described markers B2_5118, B4_4965 and B4_7686 showed a high correlation with 25 additional features according to “Pattern Hunter” a tool from MetaboAnalyst software^35^ (See Supplementary Fig. S3). This analysis allowed a putative identification of the possible compounds that corresponded to those features. In addition, PeakForest 2.0.1® software functions were used with a correlation coefficient of ±0.5 to help in the identification of metabolites.

### Confidence annotation of compound identification

Confidence annotation consisted of three steps. First, a literature review of the compounds that have been isolated from the species was performed using the SciFinder® database. Cembrane-type diterpenoids have been isolated from *Plexaurella* sp. and *P. kukenthali*^44^. From a biomedical perspective, some of those cembranes have shown cytotoxic activity against several tumor cell lines and have also been reported as anti-inflammatory, neuroprotective and antimicrobial compounds^45^.

In a second step, a verification of chemical formulas and exact masses was developed using both LC-MS and LC-MS/MS with the purpose of yielding a putative identity of the compounds. The presence of the compound asperdiol in the extract of *Plexaurella* sp., with a molecular formula C_20_H_32_O_3_ corresponding to the ion *m/z* 321.2426 [M + H], was confirmed (Supplementary Fig. S5a,b). In addition, analysis of MS/MS data fragmentation using MetFrag software^46^ matched the data for the compound asperdiol (score 7.47). On the other hand, from a high-resolution electrospray ionization mass spectrometry (HRESIMS) analysis of the extract from *P. kukenthali*, the formula C_20_H_34_O_3_ was calculated, which confirmed the possible presence of the compound plexaurolone with an ion *m/z* of 323.25860 [M + H] and an *m/z* of 345.24038 [M + Na] (Supplementary Fig. S6a,b)^47^.

In the third step, chemical shifts were analyzed using the AntiMarin® database to check signal patterns that corresponded to the kind of compounds (diterpenoid-type metabolites) expected to be present in the mixture (diterpenoid-type compounds)^48^. As a consequence, in the ^1^H-NMR spectra of the extracts from *Plexaura kukenthali* and *Plexaurella* sp. (Fig. S4 in Supplementary Information), singlets were found at a range of chemical shifts from approximately 0.5 to 2.0 ppm, indicating the presence of several methyl groups, which is characteristic of terpenoid-type compounds and in agreement with the literature^49^. Additionally, chemical signals of VIP markers B4_4965 and B4_7686 were found in the ^1^H-NMR spectrum of the extract from *Plexaurella* sp. When comparing these data with those reported in the literature, we detected similarities with the NMR data published for the antineoplastic compound asperdiol^50^. Additionally, an analysis of the marker B2_5118 in the ^1^H-NMR spectrum of the extract from *P. kukenthali* and further comparisons with NMR data reported in the literature allowed us to make a putative identification of this compound as plexaurolone. Table S5 shows the summarizing the key results of this study.

Finally, in accordance with recent discussions in the metabolomics community^51^, the confidence grade in the identification of these metabolites (asperdiol and plexaurolone shown in the Supplementary Information) is level 2 because this level reveals probable structure using fragmentation data from literature and/or libraries and databases.

## Discussion

There are two analytical techniques commonly used in metabolomics, each with advantages and disadvantages: mass spectrometry (MS) and nuclear magnetic resonance (NMR) spectroscopy. There is an increasing growth in NMR-based metabolomics over the last few years due to the advantages that this technique offers: high reproducibility and quantification ability over a wide dynamic range of compounds and support for the identification of unknown structures; moreover, NMR is nondestructive and can be utilized *in vivo*^48^.

NMR is a very suitable method for analysis that allows the simultaneous detection of diverse groups of secondary metabolites in complex matrices, as evidenced by a study conducted by Mohamed *et al*. where some cembrane diterpenoids were isolated from the *Sarcophyton ehrenbergi* soft coral and identified using NMR techniques^52^. In another study, Zhang *et al*., also using NMR, found cytotoxic diterpenoids from the soft coral *Sinularia microclavata*^53^.

One-dimensional (1D) ^1^H-NMR is the most widely used NMR approach in metabolomics. However, this approach has some limitations being key signal identification and accurate peak integration the most challenging, due to hundreds of overlapping signals^54^. These challenging conditions can be solved if the density of signals in a spectrum is decreased by almost an order of magnitude, whereby a pure shift experiment (PSYCHE) appear as an alternative that offers greatly improved signal resolution and spectral purity. Thus, it has the potential to find wide application in the NMR spectroscopy of small molecules and NMR‐based metabolomics^21,55^, as we demonstrate in this research by using the PSYCHE experiment in soft coral metabolomics for the first time.

Here, the application of OPLS-DA to 1D ^1^H-NMR data (Table 1) revealed an excessive data adjustment in the model that could be attributed to signal overlap in the ^1^H-NMR spectra of soft-coral extracts, which was principally due to olefinic protons of diterpenes and the high-field region between 3.2 and 0.8 ppm having a high density of signals due to terpene methyl, methylene, and methine resonances, revealing that soft coral extracts contain large amounts of terpenoids^56^. The noise present in each spectrum of this type of biological sample hinders the alignment and creation of the bins used to reduce the data dimensionality in the NMR spectrum from several thousand points^34^. Therefore, the results obtained from this experiment were not conclusive, which agrees with the reports by Farag *et al*. 2017.

Validations using OPLS-DA of PSYCHE NMR experiment data (Table 2), on the other hand, showed an improvement in the total amount of variance, accuracy and reproducibility of the models, particularly for the PC3 tumor cell line, which was predictive according to the validation results. This result showed that metabolomic studies using PSYCHE NMR experiments allowed us to obtain a more reliable correlation between the chemical composition and the cytotoxic activity of soft coral extracts against the PC3 tumor cell line than metabolomic studies using solely ^1^H-NMR data. The results obtained throughout the metabolomic process always showed a correlation in the data that was identified by the PCA, OPLS-DA, the markers displayed in the VIP table, and an S-plot obtained from PLS-DA of the extracts from *P. kukenthali* and *Plexaurella* sp. These organisms were highlighted in all the analyses as those responsible for the separation of the groups that presented cytotoxic activity and those indicating that the variables B2_5118, B4_4965 and B4_7686 (chemical shifts) that were present in these species were the main markers related to the activity shown; also, we can derive from this analysis that these extracts, in addition to exhibiting the greatest cytotoxicity, were the closest in the orthogonal t score, indicating they may have a common chemical composition. This was evidenced from MS and MS/MS spectra (Supplementary Figs. S5 and S6), which showed that the chemical formulas C_20_H_32_O_3_ (with an *m/z* 321.2426 [M + H]) and C_20_H_34_O_3_ (with an *m/z* of 323.25860 [M + H]) corresponded to the compounds asperdiol and plexaurolone, respectively. They also showed that both compounds were present in the two soft coral species. However, the ^1^H-NMR signals corresponding to asperdiol were not clearly observed in the extract of *P. kukenthali*, and in the same way, the chemical shifts of plexaurolone could not be observed in *Plexaurella* sp. extracts due to plexaurolone abundance.

Here, the fact that the extracts from *P. kukenthali* and *Plexaurella* sp. were mainly responsible for the separation of the group that presented cytotoxic activity was statistically validated using the cross-validation method (Table 2), which agreed with the cytotoxicity presented by these species as reported by Honda *et al*.^57^ and Rueda *et al*.^58^, who showed that some terpenoids obtained from *Plexaurella grisea* and *P. kukenthali* exhibited cytotoxic activity against myeloid leukemia and P-388 cancer cell lines.

From analyses of the VIP and the OPLS score plot (Fig. 5), it was established that the most important characteristics in the separation of extracts that showed cytotoxic activity were B2_5118, B4_4965 and B4_7686. Based on their ^1^H-NMR chemical signals, it was possible to make a putative identification of asperdiol in the extract of *Plexaurella* sp. due to the signals evident in the extract; these signals agree with the chemical shifts for this compound reported by Weinheimer and Matson (1977)^59^. Chemical shifts at 4.76 and 4.95 ppm correspond to an exomethylene group characteristic of the cembrane skeleton. Signals located at 4.50 and 4.05 ppm are characteristic of carbinolic methine and methylene groups. Additionally, three signals, 1.20, 1.62 and 1.77 ppm, were assigned to three methyl groups, and the signal located at 2.70 ppm corresponds to an epoxy proton characteristic of asperdiol. The remaining chemical shifts were consistent with those reported in the literature^50^. In addition, chemical shifts assigned to the marker B2_5118 confirmed that the compound plexaurolone was present in the extract of *P. kukenthali*, in agreement with NMR data reported for this compound^47^. The signal located at 2.53 ppm was assigned to a typical methine that supports the isopropenyl group in the cembrane skeleton. Additionally, the signals at 0.99, 1.03 and 1.04 ppm were in agreement with three methyl groups, and the signal at 4.72 ppm together with the signal 1.68 ppm were assigned to an isopropenyl group typical in this kind of nucleus. All the remaining signals were consistent with data reported in the literature^47^.

Finally, the main prospective application of metabolomic analyses using PSYCHE NMR experiments in soft corals was identifying metabolites with potential cytotoxic activity. This metabolomic approach may be useful in various scenarios but mostly in untargeted studies in which the identification of compounds is a challenge that involves a long time isolating and identifying biologically active metabolites. In addition, the advantage of using the decoupled PSYCHE experiments over the ^1^H-NMR experiments due to the overlapping of signals present in the latter is corroborated.

## Conclusion

In this study, it was possible to correlate the chemical compositions of extracts from soft coral with their cytotoxic activity against the tumor cell line PC3 using a metabolomics workflow and PSYCHE NMR experiments. By using this approach, it was possible to resolve the overlapping of ^1^H-^1^H scalar coupling multiplets, yielding an adequate matrix for reliable statistical and chemical shift analyses of complex natural products. Additionally, a preliminary identification of features responsible for the separation of the groups of extracts was possible due to the chemical shifts observed in the VIP analysis, which were deemed the most important projection variables. The PSYCHE NMR experiment, combined with metabolomics studies, allowed the development of a procedure/methodology to establish which extracts from complex biological samples were most active and allowed the identification of compounds responsible for the activity. Extracts from *P. kukenthali* and *Plexaurella* sp. were the most cytotoxic and were responsible for separation between the groups. Asperdiol and plexaurolone were the compounds responsible for the cytotoxicity exhibited by the most-active extracts.

## Materials and Methods

### Materials

Methanol and dichloromethane used for extraction were bought from Merck (Darmstadt, Germany). Cell culture reagents, D-MEM (Dulbecco’s Modified Eagle Medium (1×), RPMI 1640 Roswell Park Memorial Institute, Darmstadt, Germany were made by Gibco/Invitrogen, Paisley, UK. Other reagents were Fetal bovine serum (FBS), Eurobio brand (Les Ulis, France), trypticase soy broth (TSB) and trypticase soy agar (TSA) Scharlau Co. brand (Barcelona, Spain). All cancer cell lines were acquired from ATCC, PC3 human prostatic carcinoma (ATCC^®^ CRL1435™), SiHa human cervical carcinoma (ATCC^®^ HTB-35™) and A549 human lung adenocarcinoma (ATCC^®^ CCL-185™).

### Methods

#### Soft coral collection and identification

Small portions (30 cm) of soft corals (N = 24) (Table S2, Supplementary Information) were collected by SCUBA diving at Punta Venado (11°16.26′ 87′′N, 74°12.24′58′′W), Santa Marta, Colombian Caribbean. Samples were collected at a depth range between 10–20 m. Collected samples were stored in dry ice and transported to the laboratory. These were kept frozen at −80 °C until extraction.

Samples were identified by colony morphology and sclerite shape, dimensions and distribution. Sclerites were obtained from a distal fragment of each soft coral portion that was treated with 5% sodium hypochlorite. Once the organic material was dissolved, sclerites were observed under the microscope. A voucher of each sample is stored at the collection of the Instituto de Ciencias Naturales (ICN) of Universidad Nacional de Colombia (Bogotá, Colombia) (Table S2 in Supplementary Information).

#### General experimental procedures

All NMR data were acquired using nonspinning samples on a 600 MHz Agilent DD2 NMR at 25 °C equipped with a 5 mm C13 enhanced HCN cold probe. Signals were referenced in ppm in reference to the residual solvent signals (CDCl_3_, at δH 7.26). The ^1^H NMR spectra were acquired with 32 transients, a 1 s relaxation delay (d1), a 90-degree pulse of 6.70 µs and a 9.6 kHz (16 ppm) spectral window (sw). The transmitter offset was set to 6 ppm.

The PSYCHE (pure shift yielded by chirp excitation) spectra were acquired with 6 transients, a 1 s relaxation delay (d1), steady state scans of 2 (ss) and a tauPS of 6.3 ms, 1/sw1 = 0.12 µsec. The spectral window was set to 6 kHz (10 ppm) with the transmitter offset at 3 ppm. A wurst 180 pulse with a 6.0 deg flip angle, a 531(1.0 G/cm) gradient and a pulse width of 30.0 ms was used. A Grad-90-Grad option for the PSYCHE steady state with a G-strength of 6372 and G-time of 2 ms was used along with the Echo gradients (Encode Grad (5.0 G/cm)) using a 1.0 ms time, a 1.5 ms recovery time and a strength of 2659.

#### Sample extraction

Prior to extraction, each soft coral fragment was ground. Subsequently, 10 g of dried powder of each soft coral sample was extracted at room temperature with a mixture of solvents (DCM/MeOH, 1:1) three times (300 mL) using an ultrasonic bath for 20 minutes. Remaining debris was removed by centrifugation twice at 12,000×g for 5 minutes, the solvent was then evaporated, and the dried samples were passed through a C18 cartridge with MeOH to remove salts. Extracts were concentrated under vacuum using rotary evaporation.

#### Cytotoxicity assay

Tumor cell lines A549, SiHa and PC3 were cultured in DMEM supplemented with 10% heat-inactivated fetal bovine serum (FBS) and penicillin-streptomycin (1%) at 37 °C in a 5% CO_2_ humidified atmosphere until 100% cell confluence was achieved^46^.

The *in vitro* cytotoxicity of soft coral extracts was evaluated using the MTT method following Mosmann (1983) with modifications by Denizot and Lang (1986). This is a colorimetric assay based on the capacity of mitochondrial succinate dehydrogenase enzymes, found in living cells, to reduce the yellow, water-soluble substrate 3-(4,5-dimethyl thiazol-2-yl)-2,5-diphenyl tetrazolium bromide (MTT) into an insoluble, colored, formazan product, which is measured spectrophotometrically. Cells were grown and left to attach in 96-well plates (4.0E + 04 cells/well) for 48 hours.

A preliminary screening was performed using the 24 soft coral extracts evaluated at 20 µg/mL and DMSO at 0.1%, which was not cytotoxic to any of the cell lines. Then, the supernatant was removed, and 100 μL of 12 mM MTT solution in sterile PBS was added to each well and incubated at 37 °C for 4 hours. The solution was removed, and dimethyl sulfoxide (DMSO) was added to each well, followed by incubation at 37 °C for 15 minutes. Cell density was read in an iMarkTM Microplate Reader at a wavelength of 595 nm. Controls consisted of cells cultured without extract, and cells exposed to doxorubicin (25 ppm) were a positive control^60,61^. All tests were performed in triplicate. The cell viability percentage was calculated with Eq. (1), and the cell inhibition percentage was calculated with Eq. (2)^62^:

1 $$ \% viability=\frac{(Ab{s}_{sample})}{Ab{s}_{control}}\ast 100$$

where Abs_sample_ is the absorbance of cells treated with the test extract, and Abs_control_ is the absorbance of untreated cells.

2 $$ \% cell\,inhibition=100-Cell\,Survival$$

#### Metabolomic analyses

^1^H and PSYCHE NMR experiments of the 24 soft coral extracts were run in the NMRProcFlow v1.2 program^34^. This software performs all the spectral processing steps, including baseline correction, chemical shift calibration and alignment, and allows metabolic fingerprinting and targeted metabolomics^63^.

All spectral data were compressed with a basic Zip format and processed in the NMRProcFlow v1.2 software, which allows the input of raw data in FID format. Data processing comprises calibration of the PPM scale, baseline correction, alignment, binning and scaling.

1. Calibration of the ppm scale was performed to adjust chemical shifts according to a known reference compound. The reference compound used for the calibration of the ^1^H-NMR spectra was tetramethylsilane (TMS), with a chemical shift of δ = 0.00 ppm, and for the PSYCHE experiment, the reference compound was the solvent CDCl_3_, with a chemical shift of δ = 7.26 ppm.
2. Baseline correction was performed using the global baseline correction algorithm^64^, where the correction level was chosen as the ‘soft’ level. For increased efficiency of the method, spectral level noise ranges between 10.2 and 10.5 ppm for the ^1^H-NMR experiment and between 7.7 and 8.0 ppm for the PSYCHE experiment were considered.
3. Alignment steps were very tedious to solve. Misalignments are a result of changes in the chemical shifts of NMR peaks largely due to differences in pH, ionic strength or other physicochemical interactions^65^. To perform this step, we use the algorithm based on least squares.
4. Binning. An NMR spectrum may contain several thousands of variables. Binning is used to reduce data dimensionality. When binning, spectra are divided into bins (so-called buckets), and the total area (sum of each resonance intensity) within each bin is calculated to represent the original spectrum. The approach we chose was the adaptive ‘Intelligent Binning’ method^66^. This allowed us to split the spectra so that each area, common to all spectra, contained the same single resonance, i.e., belonged to the same metabolite.
5. Normalization. Before bucket data export, to make all spectra comparable with each other, variations in the total concentrations of samples must be considered. We used the constant sum normalization, which consists of normalizing the total intensity of each individual spectrum to the same value^67^. After the data matrices of each of the selected regions were exported, they were used for statistical analysis using MetaboAnalyst version 3.0^35^.

#### Multivariate data analysis

All variables of the data matrix were scaled using a self-scaling algorithm prior to multivariate data analysis. Principal component analysis (PCA), an unsupervised pattern recognition tool that explains the maximum amount of variation inherent in a multidimensional dataset^68^, was carried out to detect patterns in the variables matrix and to detect outliers.

Orthogonal partial least squares discriminate analysis (OPLS-DA) is a supervised pattern recognition technique that aims at finding the maximum separation between a priori established groups^69^. Therefore, OPLS-DA was applied to discriminate spectral data obtained from the ^1^H and PSYCHE NMR experiments and to discriminate between groups that exhibited or did not exhibit cytotoxicity.

The resulting models were evaluated using both R2 and Q2 metrics. R2 values reported the total amount of variance explained by the model in both the data (R2X) and independent variables (R2Y). Q2 reported the model accuracy and was calculated by cross-validation^70^.

## Supplementary information

Supplementary Information
