# Supplementary material for: Metabolomic study of soft corals from the Colombian Caribbean: PSYCHE and 1H-NMR comparative analysis
Source: Sci Rep. 2020 Mar 25;10:5417. doi: 10.1038/s41598-020-62413-0 (PMC7096504; doi:10.1038/s41598-020-62413-0)
Supplement: Supplementary file 1 — Supplementary Information [file 41598_2020_62413_MOESM1_ESM.pdf]

Metabolomic study of soft corals from the Colombian Caribbean: PSYCHE and ^{1}H NMR comparative analysis

Liliana Santacruz[1], Diana X. Hurtado[1], Roisin Doohan[2], Olivier P. Thomas[2], Mónica Puyana[3] and Edisson Tello[*1]

[1] Bioprospecting Research Group and Biosciences Doctoral Program, Faculty of Engineering, Campus Puente del Común, Universidad de la Sabana, 250001 Chía, Colombia
[2] Marine Biodiscovery, School of Chemistry and Ryan Institute, National University of Ireland Galway (NUI Galway), University Road, H91 TK33 Galway, Ireland
[3] Departamento de Ciencias Biológicas y Ambientales, Universidad Jorge Tadeo Lozano, Carrera 4 # 22-61, 110311 Bogotá, Colombia; monica.puyana@utadeo.edu.co

*****Correspondence: edisson.tello@unisabana.edu.co (E.T.)
Tel.: +57-32-0699-5696 (E.T.)

Most commonly used methods for metabolomics analysis - advantages and disadvantages

Technology	Advantages	Disadvantages
**MS (LC/MS and GC/MS)**	• High analytical sensitivity<br>• Robust technique<br>• give more information (TR, MS)	• Volume Large sample is required (500 L)<br>• sample Destructive<br>• Extensive sample preparation, (GC-MS) requires derivatization
**NMR Spectroscopy (^{1}H/PSYCHE)**	• Less sample is required (1–10 L)<br>• Robust and reproducible<br>• Non-destructive sample<br>• Minimal sample preparation<br>• Time acquisition (≈7min)	• Low analytical sensitivity<br>• Signal overlap<br>• give less information (δppm)<br>• Time acquisition (≈20 - 30min)

Table S1. Advantages and disadvantages of the most commonly used methods for metabolomics analysis

Name assigned by collection area	Name of the species	Accession	Código ICN-UN
Cali 10	*Muriceopsis flavida*	C10m	ICN-MHN (Po)-CO-292
Cali 11	*Eunicea flexuosa*	C11E	ICN-MHN (Po)-CO-276
Cali 15	*Pseudopterogorgia albatrossae*	C15Pst	ICN-MHN (Po)-CO-278
Cali 16	*Eunicea fusca*	C16E	ICN-MHN (Po)-CO-277
Cali 18	*Muriceopsis flavida*	C18m	ICN-MHN (Po)-CO-292
Cali 19	*Eunicea flexuosa*	C11E	ICN-MHN (Po)-CO-276
Cali 2	*Plexaura kukenthali*	C2P	ICN-MHN (Po)-CO-271
Cali 24	*Plexaurella nutans*	C24p	ICN-MHN (Po)-CO-281
Gra 10	*Eunicea knightii*	G10Ek	ICN-MHN (Po)-CO-287
Gra 1	*Plexaurella fusifera*	G1P Plr	ICN-MHN (Po)-CO-282
Gra 13	*Eunicea clavigera*	G13E	ICN-MHN (Po)-CO-275
Gra 14	*Eunicea cf. calyculata*	G14E	ICN-MHN (Po)-CO-288

Gra 16	*Eunicea knightii*	G16Ek	ICN-MHN (Po)-CO-287
Gra 17	*Pseudoplexaura flagellosa*	G17Ps	ICN-MHN (Po)-CO-289
Gra 18	*Plexaura kukenthali*	G18P	ICN-MHN (Po)-CO-292
Gra 21	*Plexaura homomalla*	G21P	ICN-MHN (Po)-CO-283
Gra 2	*Plexaura homomalla*	G2P	ICN-MHN (Po)-CO-283
Gra 22	*Plexaurella* sp.	G22P	ICN-MHN (Po)-CO-291
Gra 24	*Plexaura homomalla*	G2P	ICN-MHN (Po)-CO-283
Gra 3	*Eunicea clavigera*	G13E	ICN-MHN (Po)-CO-284
Gra 4	*Eunicea asperula*	G4E	ICN-MHN (Po)-CO-285
Gra 6	*Plexaura kukenthali*	G6Pk	ICN-MHN (Po)-CO-271
Gra 8	*Eunicea clavigera*	G8E	ICN-MHN (Po)-CO-275
Gra 9	*Plexaura sp.*	G9P	ICN-MHN (Po)-CO-286

Table S2. Name of species of soft corals used in this research a Collection Code (ICN), assigned by Collection of the Institute of Natural Sciences of the National University of Colombia. (Bogotá, Colombia)

Obs	name	Siha (%)	Pc3 (%)	A549 (%)	L929 (%)
1	C10m	23.1	20.8	31.0	49.8
2	C11E	36.8	12.1	49.3	21.7
3	C15Pst	26.8	20.3	71.5	25.9
4	C16E	60.5	30.0	39.1	21.3
5	C18m	21.2	15.0	26.5	13.8
6	C19E	15.1	16.1	36.1	20.7
7	C2P	46.2	7.8	62.4	16.2
8	C24p	13.1	65.1	39.0	13.3
9	G10Ek	46.1	1.3	35.4	14.5
10	G1P Plr	39.4	23.8	33.0	11.5
11	G13E	46.9	30.9	39.7	6.8
12	G14E	63.5	22.0	65.7	19.2
13	G16Ek	26.1	15.3	30.4	14.5
14	G17Ps	66.8	40.3	71.5	65.9
15	G18P	44.0	64.0	52.5	31.2
16	G21P	25.5	20.9	34.0	18.4
17	G2P	34.8	47.6	21.1	14.3
18	G22P	37.6	63.5	31.9	20.7
19	G24P	37.6	33.5	31.9	20.7
20	G3E	59.9	43.7	35.3	5.8

**21**	G4E	53.9	41.5	67.6	14.6
**22**	G6Pk	57.0	40.3	38.5	23
**23**	G8E	54.8	47.6	22.1	14.3
	G9P	34.5	20.4	31.0	49.8
**29**	Doxorubicin (25ppm)	60.1	46.9	58.2	16.0

Table S3. Percentage of cytotoxic activity against three cancerous cell lines and fibroblasts L929 (ATCC®CCL-1 ™) which was used as non-tumor cell line for toxicity control.

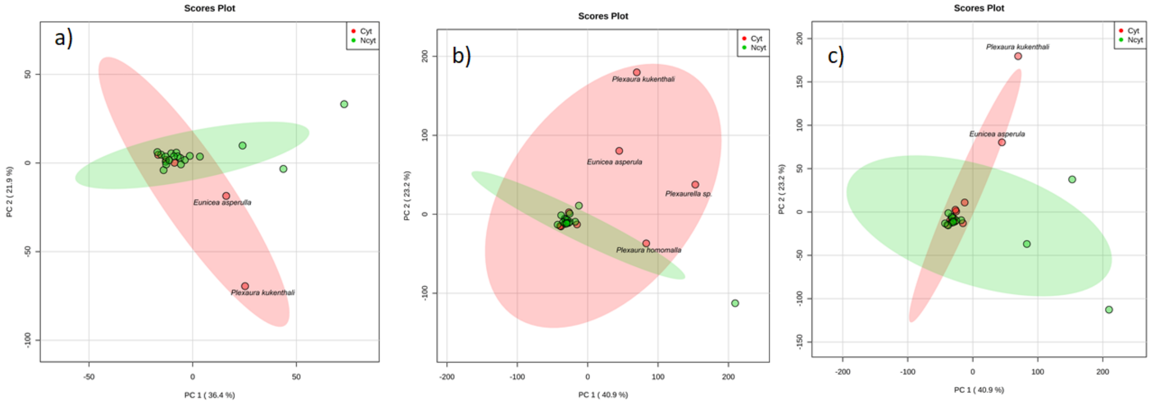

Figure S1. Principal component analysis score plot of ^{1}HNMR/metabolomics data from 24 extracts based on their cytotoxicity against different cancer cell lines (a) human lung adenocarcinoma, A549 (b) human prostatic carcinoma, PC3 and (c) human cervical cancer, SiHa. Red dots indicate active extracts, green dots represent extracts that were not active. An extract was considered active if it exhibited an inhibition of the tumor cell lines ≥ 40% at 20 µg/mL) [22]. The ellipses indicate confidence intervals of 95%.

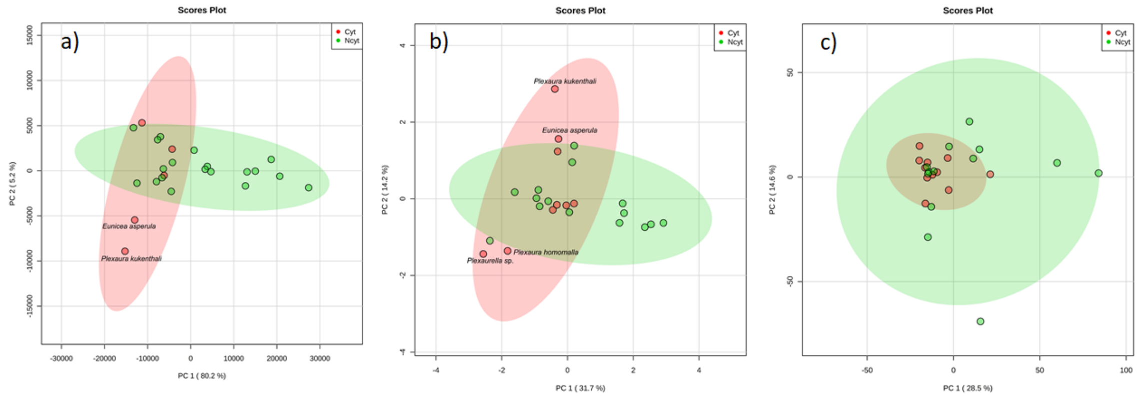

Figure S2. Principal component analysis score plot of PSYCHE/metabolomics data from 24 extracts based on their cytotoxicity against three different tumor cancer cell lines (a) human lung adenocarcinoma A549 (b) human prostatic carcinoma PC3 and (c) human cervical cancer SiHa. Red dots indicate active extracts, green dots represent extracts that were not active. An extract was considered active if it exhibited an inhibition of the tumor cell lines ≥ 40% at 20 µg/mL(Hostettman, 1991). The ellipses indicate confidence intervals of 95% (Chong et al., 2018).

Supplementary text for 1H-NMR and PSYCHE experiments data extraction

Instrument/Vendor/Format = Varian
Spectra type = fid
The original name of the Zip file = testPscomple31hasta8ppm sincali8nigra20.zip
The number of Spectra = 24
Calibration: PPM REF = 0, Zone Ref = (0.045,0.071) to PSYCHE experiment
calibration 0.045 0.071 0 10.2 10.5
Normalisation (CSN) of the Intensities based on the selected PPM ranges 0.348 7.847
Global Baseline Correction: PPM Range = (-0.499764773120937, 10.999765269097)

gbaseline 10.2 10.5 -0.499764773120937 10.9997652690972 100 70
Alignment of the selected zones (0.18,7.972)
Rbuc1D: --- BUCKETING MODULE ---
Rbuc1D: Read the SpecProcpar.ini file ...
Rbuc1D: Read the spectra processing parameters file (list_pars.csv) ...
Rbuc1D: Read the specs.pack.
Rbuc1D: Bucketing method = AIBIN
Rbuc1D: Noise Zone= (10.5 ,10.2), (818 - 1310)
Rbuc1D: Factor Resolution = 0.3
Rbuc1D: Zone 1 = (0.133, 7.905), Nb Buckets = 1230
Rbuc1D: Total Buckets = 113 to PSYCHE experiment
The parameters for 1H-NMR are the same, only the calibration varies using CDCl3:
Calibration: PPM REF = 7.26, Zone Ref = (7.25, 7.26)

Table S4. Scrip using NMRProcFlow software to 1H-NMR and PSYCHE experiments to data extraction

Main results of the study					
**Experiment**	Tumoral cell line	Cytotoxic activity (statistically significant)	Extracts that exhibit activity	VIP Features	Compounds / Putative identification
^{1}**H NMR**	**A549**	No	-	-	-
	**PC3**	No			
	**SiHa**	No	-	-	-
**Psyche**	**A549**	No			
	**PC3**	Si	• *Plexaura kukenthali*<br>• *Plexaurella* sp.	• *B2_5118*<br>• *B4_4965*<br>• *B4_7686*	• Asperdiol<br>• Plexaurolone
	**SiHa**	No	-	-	-

Table S5. Summarizing the key results of the study

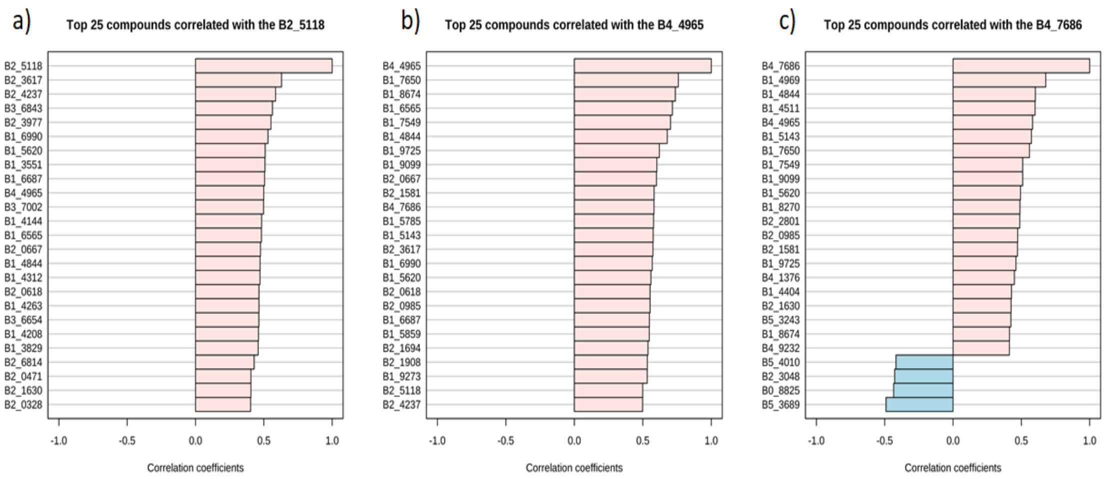

Figure S3. Twenty five features that most correlate with the features B2_5118, B_4965 and B4_7686 , established using "Pattern Hunter" a tool of MetaboAnalyst software (Chong et al., 2018).

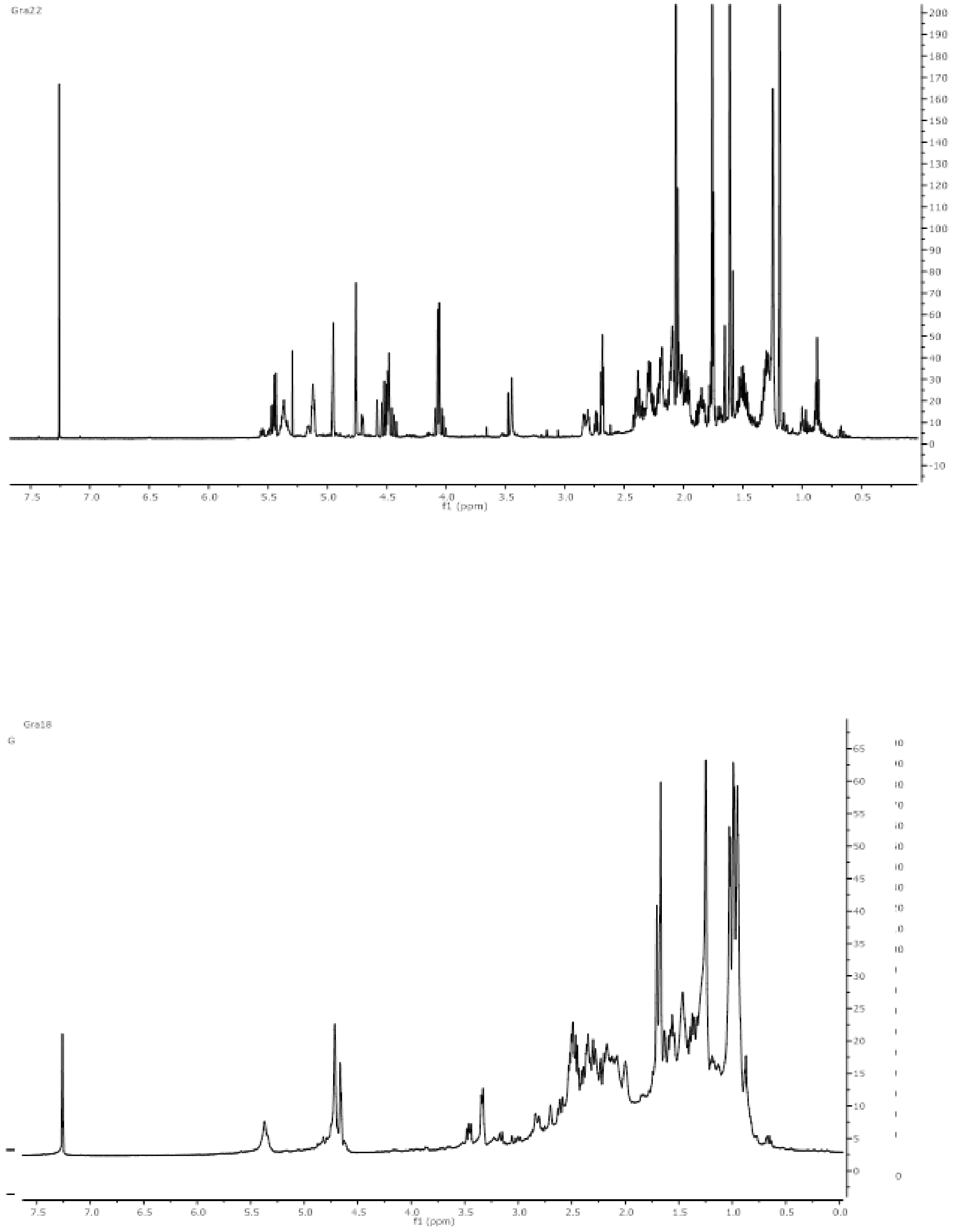

Figure S4. a) ^{1}H NMR spectrum of crude extract from specie *Plexaurella* sp, (Gra22) in $CDCl_3$ at 600 MHz. b) ^{1}H NMR spectrum of crude extract from specie *Plexaura kukenthali* (Gra18) in $CDCl_3$ at 600 MHz.

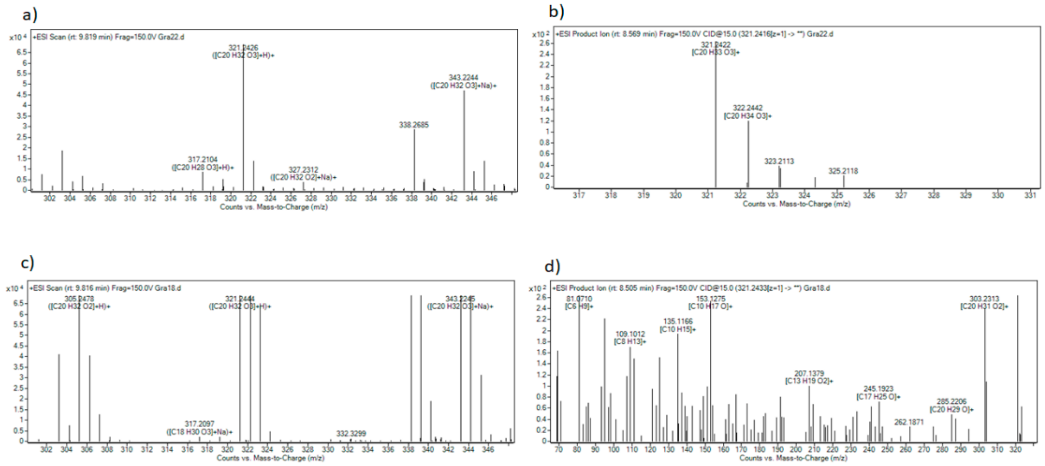

e)

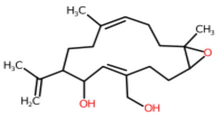

Figure S5. a) MS from *Plexaurella* sp (Gra 22) extract, b) MS/MS spectrum of 321.2416 ion from *Plexaurella* sp, c) MS from *Plexaura kukenthali* (Gra 18) extract, d) MS/MS spectrum of 321.2433 ion from *Plexaura kukenthali*. These spectrums confirming the possible presence of the compound asperdiol showing a molecular formula of $C_{20}H_{32}O_3$, e) asperdiol compound structure.

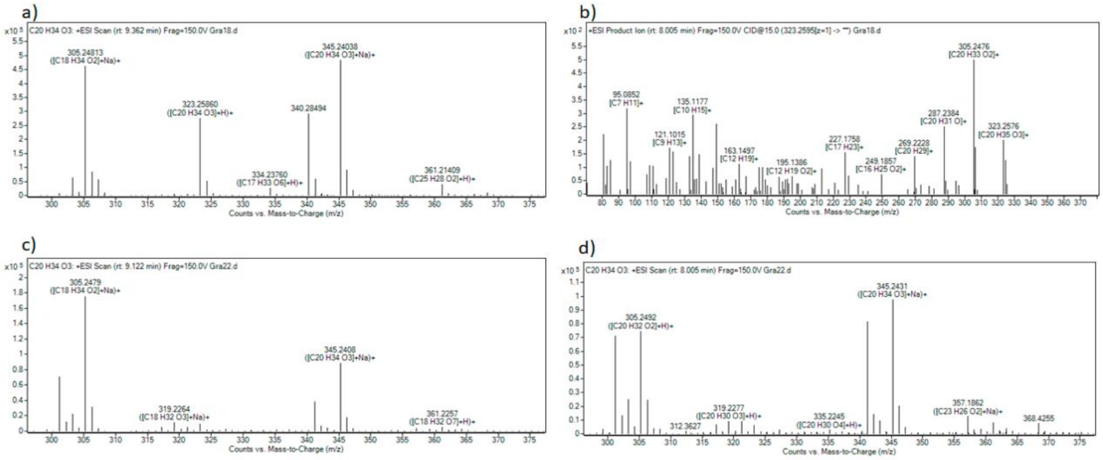

e)

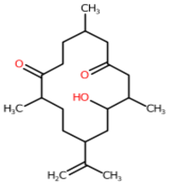

Figure S6. a) MS from *Plexaura kukenthali* (Gra 18) extract, b) MS/MS spectrum of 323.2595 ion from *Plexaurella* sp, c) MS from *Plexaurella* sp, (Gra 22) extract, d) MS/MS spectrum of 345.2431 ion from *Plexaurella* sp. These spectrums confirming the possible presence of the compound plexaurolone showing a molecular formula of $C_{20}H_{34}O_3$, e) plexaurolone compound structure.
